# Heterologous expression of the maize transcription factor ZmbHLH36 enhances abiotic stress tolerance in *Arabidopsis*

**DOI:** 10.1007/s42994-024-00159-3

**Published:** 2024-05-13

**Authors:** Zhenggang Dai, Keyong Zhao, Dengyu Zheng, Siyu Guo, Huawen Zou, Zhongyi Wu, Chun Zhang

**Affiliations:** 1 College of Agriculture, Yangtze University, Jingzhou, 434025 China; 2 Institute of Biotechnology, Beijing Academy of Agriculture and Forestry Sciences, Beijing Key Laboratory of Agricultural Gene Resources and Biotechnology, Beijing, 100097 China

**Keywords:** Heterologous expression, Maize, Root system, Stress responses, *ZmbHLH36*

## Abstract

Basic helix-loop-helix (bHLH) transcription factors are widely distributed in eukaryotes, and in plants, they regulate many biological processes, such as cell differentiation, development, metabolism, and stress responses. Few studies have focused on the roles of bHLH transcription factors in regulating growth, development, and stress responses in maize (*Zea mays*), even though such information would greatly benefit maize breeding programs. In this study, we cloned the maize transcription factor gene *ZmbHLH36* (Gene ID: 100193615, GRMZM2G008691). ZmbHLH36 possesses conserved domains characteristic of the bHLH family. RT-qPCR analysis revealed that *ZmbHLH36* was expressed at the highest level in maize roots and exhibited different expression patterns under various abiotic stress conditions. Transgenic *Arabidopsis* (*Arabidopsis thaliana*) plants heterologously expressing *ZmbHLH36* had significantly longer roots than the corresponding non-transgenic plants under 0.1 and 0.15 mol L^−1^ NaCl treatment as well as 0.2 mol L^−1^ mannitol treatment. Phenotypic analysis of soil-grown plants under stress showed that transgenic *Arabidopsis* plants harboring *ZmbHLH36* exhibited significantly enhanced drought tolerance and salt tolerance compared to the corresponding non-transgenic plants. Malondialdehyde contents were lower and peroxidase activity was higher in *ZmbHLH36*-expressing *Arabidopsis* plants than in the corresponding non-transgenic plants. ZmbHLH36 localized to the nucleus when expressed in maize protoplasts. This study provides a systematic analysis of the effects of ZmbHLH36 on root growth, development, and stress responses in transgenic *Arabidopsis*, laying a foundation for further analysis of its roles and molecular mechanisms in maize.

**Supplementary Information:**

The online version contains supplementary material available at 10.1007/s42994-024-00159-3.

## Introduction

Plants initiate a series of stress responses when facing adverse conditions, such as drought, high temperature, salinity, and other stresses (Gong et al. 2020). Transcription factors play crucial roles in these adaptive responses by regulating the expression of stress-related genes, thereby helping plants acclimate to harsh environments (Baillo et al. 2019). Numerous stress-related transcription factors have been investigated in plants (Erpen et al. 2018). These transcription factors regulate stress-responsive genes by binding to specific elements in their promoters. For example, AP2/ERF transcription factors bind to dehydration response elements (DREs/CRTs) (Xie et al. 2019), WRKY transcription factors to W-box sequences (Van Aken et al. 2013), NAC transcription factors to NAC response elements (Tran et al. 2004), MYB transcription factors to MYB binding sites (Baldoni et al. 2015), and NF-Y transcription factors to CCAAT sequences (Zhang et al. 2023).

The basic helix-loop-helix (bHLH) transcription factor family is widely conserved in eukaryotes. bHLH transcription factors have a bHLH domain containing an N-terminal basic region and a C-terminal HLH region. The basic region, typically located at the N terminus, is composed of a stretch of basic amino acids and is crucial for DNA binding and transcriptional activation. The C-terminal HLH region consists of two α helices and a short loop. One of the α helices binds to DNA and recognizes specific DNA sequences, while the other α helix interacts with other proteins, forming dimers or multimeric complexes (Hao et al. 2021).

The bHLH transcription factors represent one of the largest transcription factor families in plants, where they play important roles in signal transduction and the regulation of growth, development, and responses to environmental stress (Guo et al. 2021). For example, in *A. thaliana*, bHLH57, together with the abscisic acid (ABA)–related protein REDUCED DORMANCY 1 (AtRDO1), inhibits the expression of *NCED*, which encodes a key enzyme involved in ABA biosynthesis, thereby breaking seed dormancy (Liu et al. 2020). The phytochrome-interacting factor (PIF) photoreceptors are bHLH proteins that integrate external and internal signals by interacting with various factors to regulate seed germination, phytohormonal crosstalk, and associated signaling pathways (Pham et al. 2018). Four bHLH proteins (FBH1, FBH2, FBH3, and FBH4) act as transcriptional activators of the *CONSTANS* gene, a key factor that converts the biological clock signal into a flowering signal (Ito et al. 2012). bHLH transcription factors also regulate the growth and development of plant roots. In rice (*Oryza sativa*), a bHLH transcription factor mutant, *Osrhl1-1*, had shorter root hairs than the corresponding non-transgenic plants under low-temperature conditions (Ding et al. 2009). Exogenous overexpression of the sorghum (*Sorghum bicolor*) gene *SbbHLH85* in *Arabidopsis* led to a significant increase in root hair formation in the corresponding non-transgenic plants and restored the root hair defects in single and double mutants (Song et al. 2022).

The bHLH transcription factors also play important roles in plant resistance to abiotic stress. *Arabidopsis* plants overexpressing *bHLH122* had significantly higher ABA levels and exhibited greater tolerance to drought, salt, and osmotic stress compared to non-transgenic plants (Liu et al. 2014). Heterologous overexpression of the wheat (*Triticum aestivum*) *TabHLH1* gene enhanced the tolerance of transgenic tobacco (*Nicotiana tabacum*) to drought and salt stress by upregulating the ABA signaling genes *PYR1/PYL/RCAR1-LIKE 12* (*NtPYL12*) and *SUCROSE NON-FERMENTING1-RELATED PROTEIN KINASE 21* (*NtSAPK21*), as well as regulating intracellular substance accumulation and reactive oxygen species (ROS) homeostasis (Yang et al. 2016). Heterologous overexpression of *CgbHLH001* from *Chenopodium glaucum* (*Oxybasis glauca*) enhanced drought tolerance in maize. Under drought stress, maize plants expressing *CgbHLH001* showed increased soluble sugar and proline accumulation, increased antioxidant enzyme (superoxide dismutase [SOD], peroxidase [POD], and catalase [CAT]) activity, and upregulated expression of stress-related genes, demonstrating a drought-tolerant phenotype (Li et al. 2010). Overexpressing the bHLH family transcription factor gene *ZmPTF1* in maize increased root length, root hair quantity, and root surface area, enhancing the plant’s water absorption capacity and drought tolerance (Li et al. 2019). A signaling pathway involving the transcription factor ZmbHLH32, the Aux/IAA family gene *ZmIAA9*, and AUXIN RESPONSE FACTOR 1 (ZmARF1) positively regulates salt tolerance and inhibits the expression of ROS scavenging genes in maize (Yan et al. 2023). In rice, the cold-induced bHLH transcription factor INDUCER OF CBF EXPRESSION 1 (OsICE1), positively regulates cold tolerance in rice seedlings (Liu et al. 2023). Heterologously overexpressing the rice bHLH transcription factor gene *OrbHLH001*, which has high sequence similarity to *ICE1*, enhanced the freezing and salt tolerance of the model plant *Arabidopsis* (Chen et al. 2013; Li et al. 2010). In maize, ZmICE1 in maize plays a crucial role in regulating the expression of amino acid metabolism genes, thereby affecting cold tolerance (Jiang et al. 2022).

Although previous studies have investigated the molecular mechanisms of various bHLH transcription factors, little research has been conducted on bHLH family transcription factors in the monocotyledonous model crop plant maize. We previously performed root phenotype analysis and transcriptome sequencing (RNA-seq) of maize at key stages of growth, including the 6-leaf stage (V6), 12-leaf stage (V12), tasseling stage (VT), and milk-mature stage (R3). *ZmbHLH36* was highly expressed during periods of vigorous root growth, such as the V6 and V12 stages, pointing to its potential involvement in regulating root growth and development (Zhang et al. 2020). In this study, to elucidate the roles of ZmbHLH36 in regulating root growth and development as well as plant responses to stress, we cloned the *ZmbHLH36* gene. Using heterologous expression in *Arabidopsis*, we initiated a functional analysis of the roles of this transcription factor in root development and stress tolerance. Our findings lay a foundation for further in-depth investigation of the biological functions of ZmbHLH36 in maize.

## Results

### Cloning and structural analysis of the maize transcription factor gene *ZmbHLH36*

**Fig. 1:**
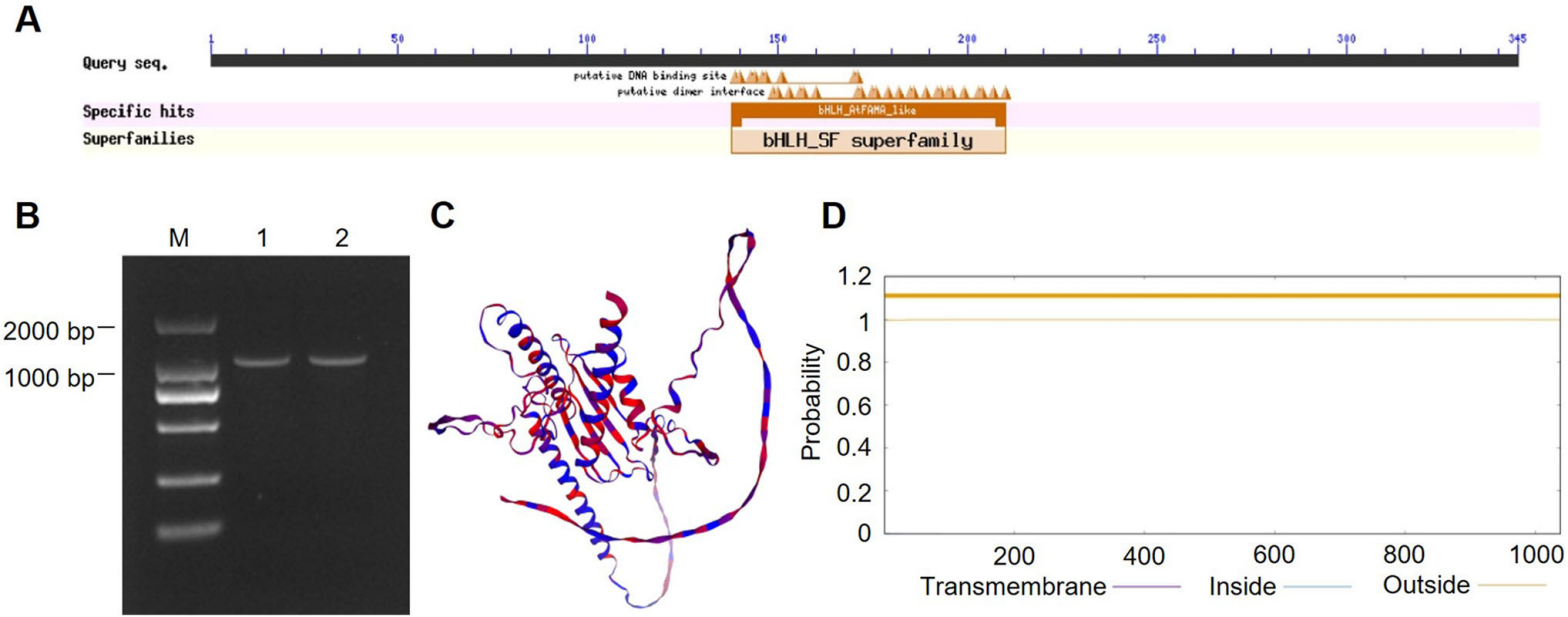
Cloning and bioinformatic analysis of the *ZmbHLH36* gene. **A** Structural domain analysis of ZmbHLH36. **B** Cloning of *ZmbHLH36*. **C** Predicted tertiary structure of ZmbHLH36. **D** Predicted transmembrane domains of ZmbHLH36

Structural domain analysis of the predicted protein encoded by *ZmbHLH36* revealed the presence of a conserved domain characteristic of the bHLH family (Fig. 1A). We amplified the coding sequence (CDS) of *ZmbHLH36* by RT-PCR. The full-length CDS was found to be 1038 bp long, encoding a protein of 345 amino acids (Fig. 1B). Analysis of the physicochemical properties of the deduced ZmbHLH36 protein sequence revealed a molecular weight of 36.39 kDa, a molecular formula of C_1567_H_2548_N_486_O_486_S_13_, and a theoretical isoelectric point (pI) of 8.59.

In the predicted secondary structure of ZmbHLH36, α-helixes comprised the highest proportion of structures (48.99%), followed by irregular coils (37.68%). The predicted tertiary structure of ZmbHLH36 is shown in Fig. 1C, and the amino acid sequence contains no predicted transmembrane structures (Fig. 1D). Analysis of the 2-kb sequence upstream of the start codon (ATG) of *ZmbHLH36* revealed the presence of potential cis-acting elements in the promoter region, including an ABRE (involved in the ABA response), a CAT-box (cis-regulatory element associated with organ-specific expression), a CGTCA-motif (jasmonic acid methyl ester response cis-regulatory element), a G-box (responsive to ABA, light, and UV radiation), a TGA-element (auxin response cis-regulatory element), and a low-temperature response element, among others.

### The expression of *ZmbHLH36* in different maize tissues

**Fig. 2:**
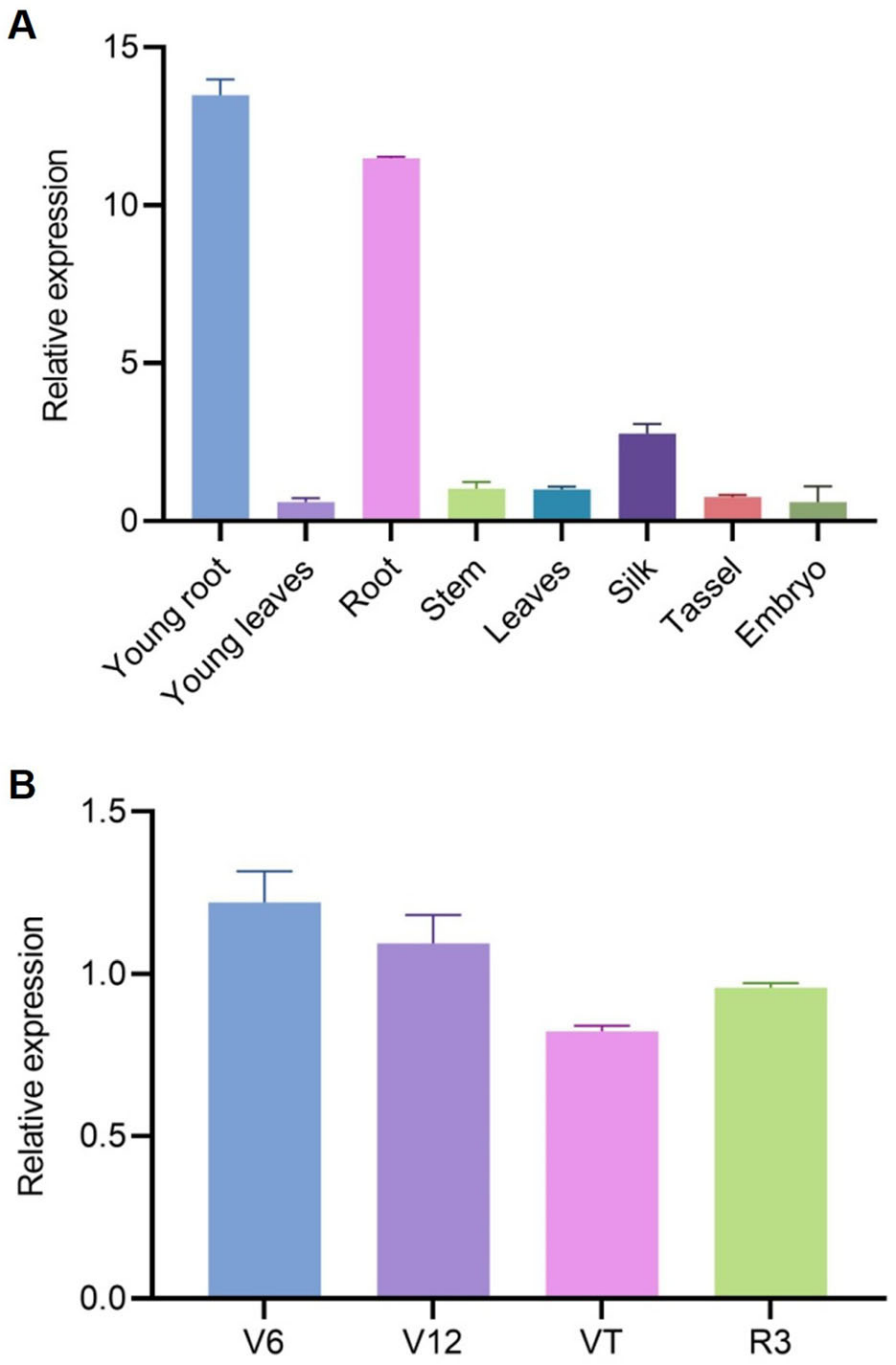
Analysis of *ZmbHLH36* expression in different tissues and during key stages of maize root growth and development. **A** Tissue-specific expression of *ZmbHLH36* determined by reverse-transcription quantitative PCR (RT-qPCR). The analyzed tissues included young roots and leaves; roots, stems, leaves, male inflorescences, and stamens at the tassel stage; and young embryos 1 week after pollination. **B** Relative expression levels of *ZmbHLH36* in maize roots at growth stages V6, V12, VT, and R3. Data are means ± SD (Student’s *t* tests, *P < 0.05, **P < 0.01, *n* = 3 biological replicates)

We examined the relative expression of *ZmbHLH36* in various maize tissues, including young roots and leaves of plants at the three-leaf stage; roots and leaves of plants at the tassel stage; and silks, tassels, and young embryos 10 days after pollination, via reverse-transcription quantitative PCR (RT-qPCR). The expression level in mature leaves was set to 1. *ZmbHLH36* was expressed in all eight tissues examined, with higher expression in roots than in the other tissues, particularly in young roots of plants at the three-leaf stage (Fig. 2A). The V6 and V12 stages represent periods of active root growth and development. The expression level of *ZmbHLH36* was higher in the V6 and V12 stages compared to VT and R3 (Fig. 2B). These results suggest that ZmbHLH36 may be involved in regulating root growth and development.

### Expression of *ZmbHLH36* under different abiotic stresses

**Fig. 3:**
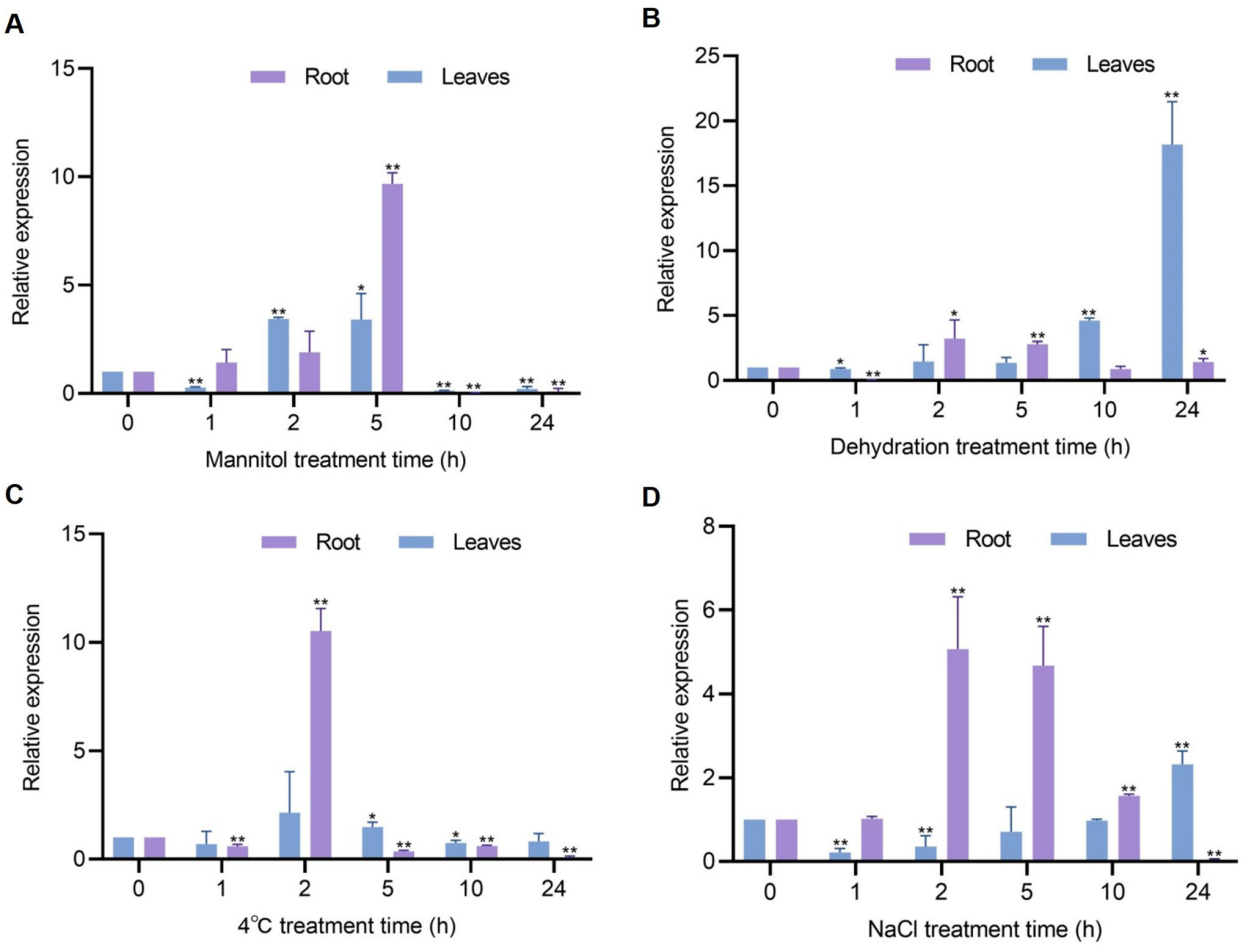
Relative expression level of *ZmbHLH36* under different abiotic stress conditions. **A–D** The expression pattern of *ZmbHLH36* in maize roots and leaves under **A** 0.2 mol L^−1^ mannitol treatment, **B** dehydration treatment, **C** 4 °C treatment, and **D** 0.2 mol L^−1^ NaCl treatment. Three independent biological experiments were conducted using nine individual maize seedlings at the three-leaf stage. The seedlings were subjected to 0.2 mol L^−1^ NaCl, 0.2 mol L^−1^ mannitol, 4 °C, or natural dehydration treatment for the specified time periods. Leaves and roots were collected for RNA extraction and RT-qPCR analysis. Data are means ± SD (Student’s *t* tests, *P < 0.05, **P < 0.01, *n* = 3 biological replicates)

Under osmotic stress, induced by mannitol treatment (Fig. 3A), *ZmbHLH36* was upregulated in roots, reaching its highest level of expression, approximately 9.67-fold higher than the control, at 5 h. Subsequently, *ZmbHLH36* expression sharply decreased, reaching approximately 0.1-fold that of the control at 24 h. In leaves, the expression level of *ZmbHLH36* showed an initial increase, followed by a decrease, with the highest level observed at 2 h, approximately 3.43-fold higher than the control. Under dehydration treatment (Fig. 3B), the expression of *ZmbHLH36* in leaves reached its highest peak at 24 h (approximately 20.5-fold higher than the control). The expression level in roots showed an initial increase, followed by a decrease, under this treatment.

Under cold treatment (Fig. 3C), the expression of *ZmbHLH36* in roots slightly decreased, followed by an increase at 2 h, reaching approximately 10.53-fold higher than the control. *ZmbHLH36* expression subsequently decreased, reaching its lowest value at 24 h (approximately 0.07-fold of the control). In leaves, the expression level increased at 2 h (approximately 2.15-fold higher than the control) and then decreased under this treatment. Under salt treatment (Fig. 3D), the expression of *ZmbHLH36* in roots showed an initial increase, followed by a decrease. The highest expression levels were observed at 2 and 5 h, reaching approximately 5.07- and 4.68-fold higher than the control, respectively. Subsequently, *ZmbHLH36* expression decreased to its lowest level at 24 h (approximately 0.06-fold of the control). In leaves, *ZmbHLH36* expression decreased at 1 h of salt treatment to a level approximately 0.21-fold that of the control and then increased (approximately twofold) at 24 h under this treatment. In summary, these changes in *ZmbHLH36* expression indicate that ZmbHLH36 may be involved in plant responses to various abiotic stresses, including osmotic stress, dehydration, low temperatures, and salt stress.

### The subcellular localization of ZmbHLH36 in maize protoplasts

To examine the subcellular localization of ZmbHLH36, we fused the coding sequence to the fluorescent marker protein enhanced green fluorescent protein (EGFP) and expressed the resulting fusion from the cauliflower mosaic virus 35S promoter in the construct pYBA1132-*ZmbHLH36*-*EGFP*. We introduced pYBA1132-*ZmbHLH36*-*EGFP* into protoplasts from etiolated seedlings of maize inbred line Z58 using polyethylene glycol–Ca^2+^-mediated transfection. The pYBA1132-*EGFP* empty vector was used as a negative control. After 12 h of cultivation in the dark, protoplasts were observed under a confocal laser-scanning fluorescence microscope (Fig. S1). Protoplasts harboring the pYBA1132-*EGFP* empty vector exhibited green fluorescence throughout the entire cell, while protoplasts harboring the pYBA1132-*ZmbHLH36*-*EGFP* vector showed green fluorescent signals only in the nucleus, indicating that ZmbHLH36 localizes to the nucleus.

### Heterologous expression of *ZmbHLH36* in transgenic *Arabidopsis* confers tolerance to salt, mannitol, ABA, and jasmonic acid

**Fig. 4:**
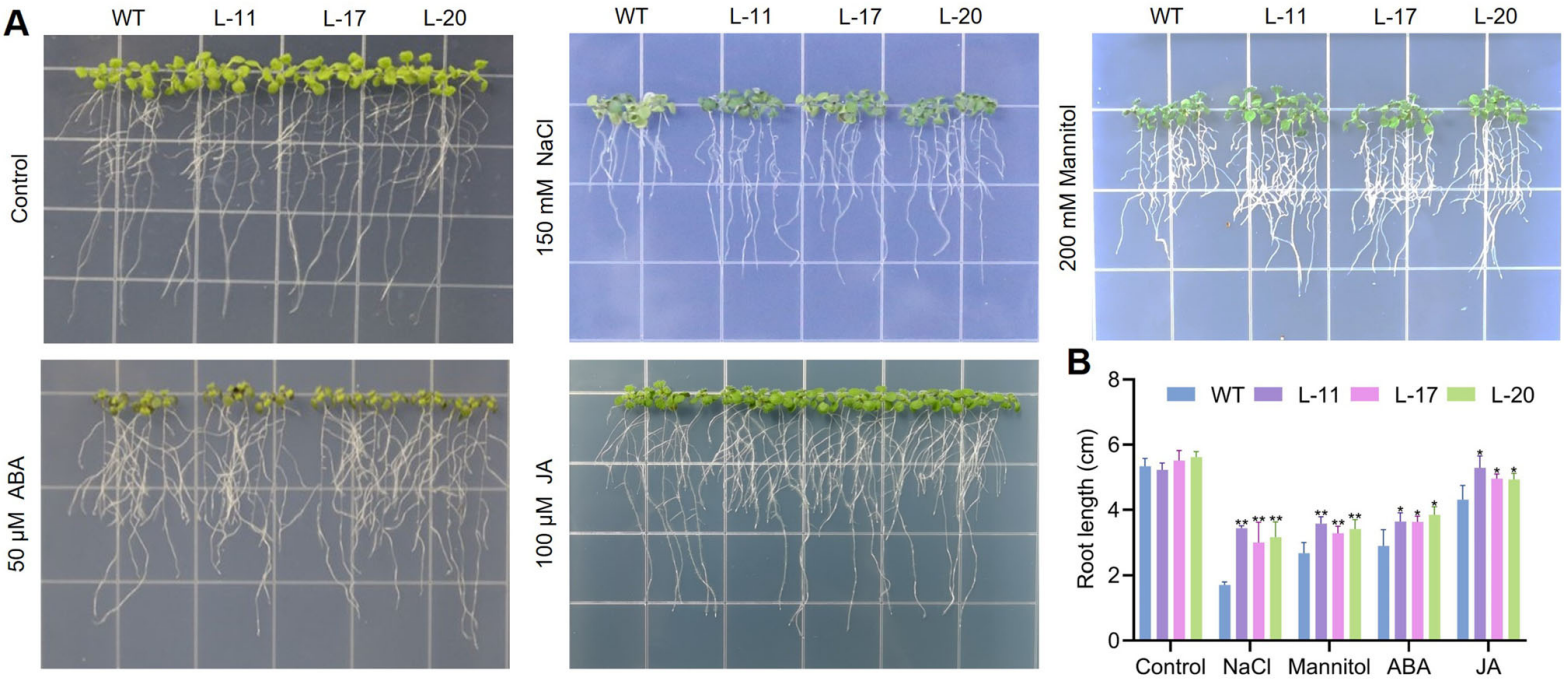
Phenotypic analysis of WT and transgenic *Arabidopsis* plants under NaCl, mannitol, ABA, and JA treatments. **A** Transgenic and WT seedlings were cultured on 1/2 MS medium supplemented with 150 mM NaCl, 200 mM mannitol, 50 μM ABA, or 100 μM JA, as well as 1/2 MS medium without any additional additives. **B** Primary root length of plants on different media; data represent average root length. Data are means ± SD (Student’s *t* tests, *P < 0.05, **P < 0.01, *n* = 15, bar = 1.5 cm)

To evaluate the role of *ZmbHLH36* in abiotic stress responses, we generated *Arabidopsis* plants exogenously overexpressing this gene. We grew T_3_ generation transgenic *Arabidopsis* plants (L-11, L-17, and L-20) identified by PCR and RT-qPCR alongside control, non-transgenic seedlings on 1/2 MS medium containing various supplements (Fig. S2). The control and transgenic plants showed similar root lengths under normal conditions. However, the roots of the transgenic plants were significantly longer than those of control plants under 150 mM NaCl or 200 mM mannitol treatment. Since bHLH transcription factors are also involved in the crosstalk among plant hormone signaling pathways, we subjected the transgenic *Arabidopsis* plants to 50 μM ABA or 100 μM jasmonic acid (JA) treatment. The roots of the transgenic plants were significantly longer than those of control plants (Fig. 4). These results suggest that ZmbHLH36 is involved in plant responses to stresses such as salt and osmotic stress and may have a regulatory role in signal transduction pathways of phytohormones such as ABA and JA.

### Drought tolerance of *ZmbHLH36* transgenic *Arabidopsis* in soil

**Fig. 5:**
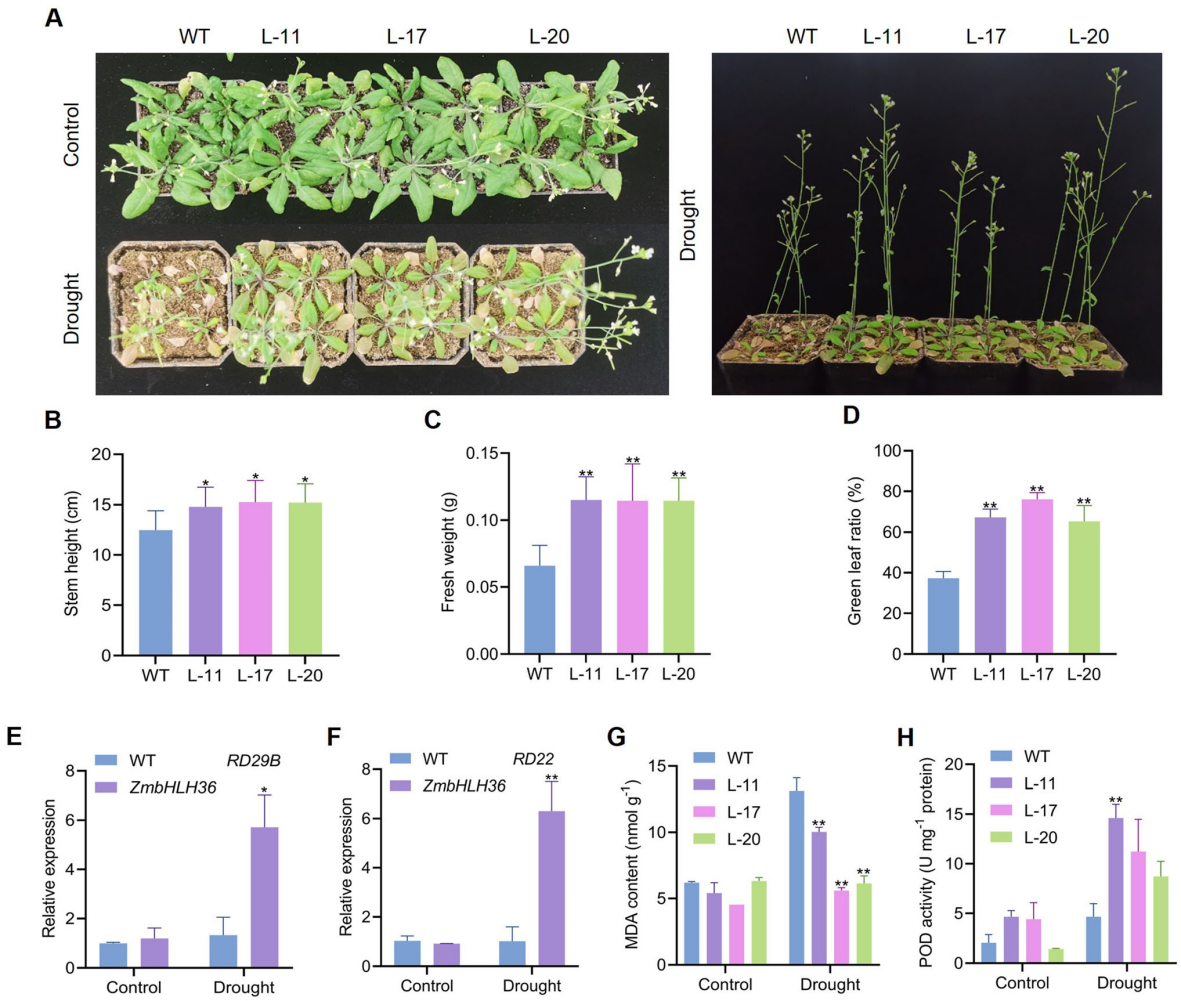
Phenotypic analysis of transgenic *Arabidopsis* under drought treatment in soil. **A** Phenotypes of *Arabidopsis* plants. **B** Plant height (Student’s *t* tests, *P < 0.05, *n* ≥ 12). **C** Shoot fresh weight (Student’s *t* tests, **P < 0.01, *n* ≥ 12). **D** Green leaf ratio under drought treatment (Student’s *t* tests, **P < 0.01, *n* ≥ 12). **E** Quantification of expression of the marker gene *RD29B*. **F** Quantification of expression of the marker gene *RD22*. **G** MDA content. **H** POD activity. Abbreviations are the same as in Fig. 4. Data are means ± SD (Student’s *t* tests, *P < 0.05, **P < 0.01, *n* = 3 biological replicates)

Under normal conditions in soil, both *ZmbHLH36* transgenic and control *Arabidopsis* plants exhibited healthy growth with similar vigor. However, when water was withheld from the plants after 2 weeks of growth under normal conditions, the growth of the controls was significantly inhibited, and the control plants were more wilted and had smaller leaves compared to the transgenic plants (Fig. 5A). The transgenic plants exhibited significantly greater stem height, fresh weight, and green leaf ratio than control plants (Fig. 5B-D).

To further investigate the regulatory pathway of ZmbHLH36 under abiotic stress, we analyzed the expression patterns of the stress/ABA-responsive genes *RESPONSE TO DEHYDRATION 29A* (*RD29A*) (Msanne et al. 2011), *RD29B* (Nakashima et al. 2006), *RD22* (Abe et al. 1997), and *RESPONSIVE TO ABA 18* (*RAB18*) (Tripathy et al. 2021) in WT and *ZmbHLH36* transgenic plants by RT-qPCR. Under drought stress, the expression of *RD29B* and *RD22* was promoted in transgenic *Arabidopsis* plants overexpressing *ZmbHLH36* (Fig. 5E and F). Physiological measurements indicated that the transgenic *Arabidopsis* plants contained reduced malondialdehyde (MDA) contents, indicating reduced oxidative stress, and increased POD activity compared to the non-transgenic control (Fig. 5G and H). These findings demonstrate that heterologously overexpressing *ZmbHLH36* enhanced the drought tolerance of *Arabidopsis* plants.

### Salt tolerance of transgenic *Arabidopsis* expressing *ZmbHLH36* in soil

**Fig. 6:**
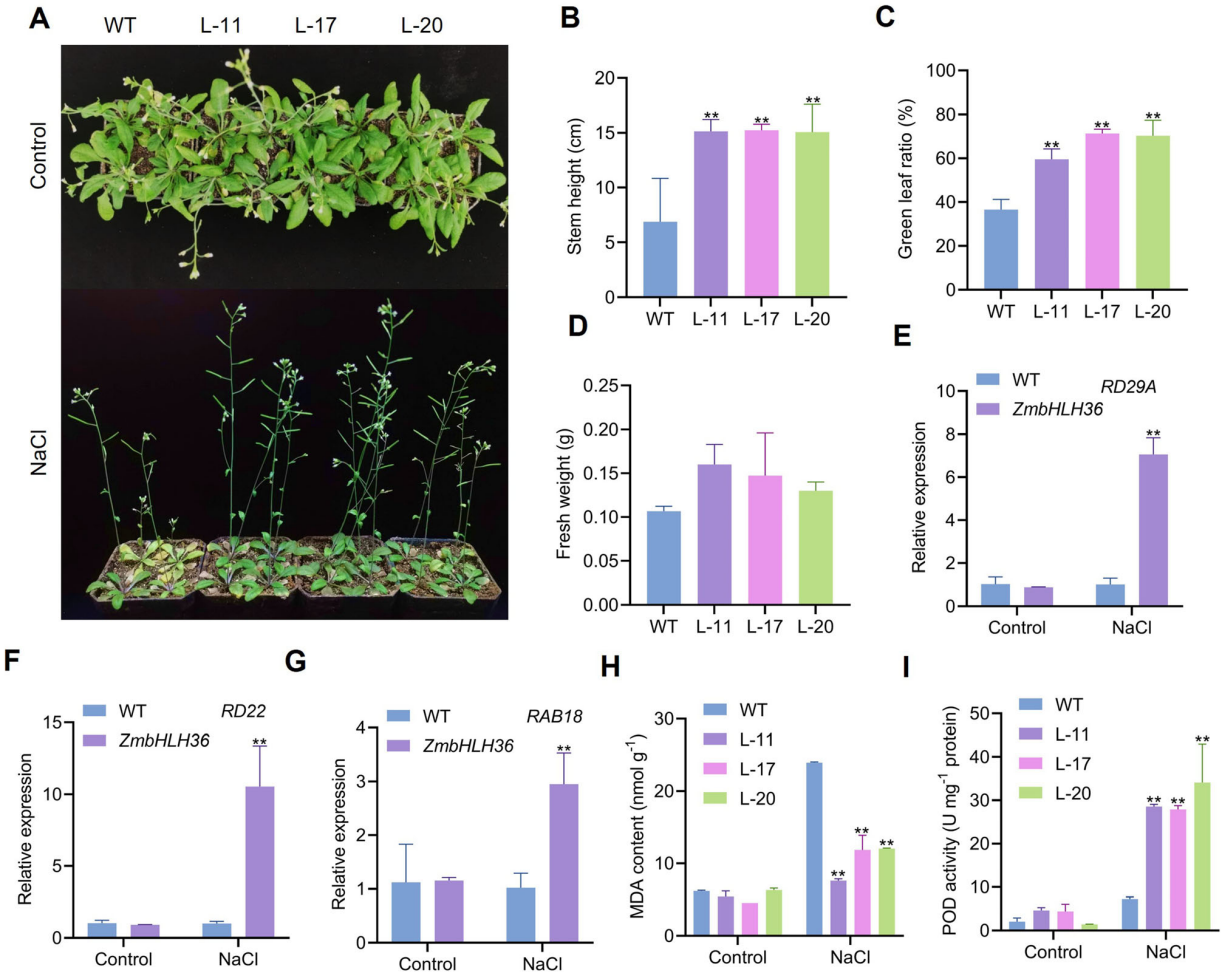
Phenotypic analysis of transgenic *Arabidopsis* under high-salt treatment in soil. **A** Phenotypes of *Arabidopsis* plants. **B** Plant height (Student’s *t* tests, **P < 0.01, *n* ≥ 12). **C** Green leaf ratio under drought treatment (Student’s *t* tests, **P < 0.01, *n* ≥ 12). **D** Shoot fresh weight (Student’s *t* tests, **P < 0.01, *n* ≥ 12). **E** Quantification of expression of the marker genes *RD29A*, **F** *RD22*, and **G** *RAB18*. **H** MDA content. **I** POD activity. Abbreviations are the same as in Fig. 4. Data are means ± SD (Student’s *t* tests, *P < 0.05, **P < 0.01, *n* = 3 biological replicates)

We analyzed the salt tolerance of transgenic *Arabidopsis* expressing *ZmbHLH36* by subjecting soil-grown plants to salt stress (300 mM NaCl) for 1 week. Under salt stress conditions, the non-transgenic control *Arabidopsis* plants exhibited symptoms such as leaf yellowing and delayed growth (Fig. 6A). By contrast, the transgenic plants showed significantly greater stem height and green leaf ratio compared to the control (Fig. 6B and C). Additionally, the transgenic plants had higher shoot fresh weight than the control (Fig. 6D). RT-qPCR analysis of stress-responsive marker genes revealed that the expression of *RD29A*, *RD22*, and *RAD18* was promoted in transgenic *Arabidopsis* plants expressing *ZmbHLH36* under salt stress but not under normal conditions (Fig. 6E-G). Physiological measurements indicated that the transgenic *Arabidopsis* plants had reduced MDA contents and increased POD activity compared to the WT (Fig. 6H and I). Collectively, these results demonstrate that exogenously expressing *ZmbHLH36* enhanced the salt tolerance of *Arabidopsis* plants.

## Discussion

bHLH transcription factors are widely distributed in eukaryotes and participate in the regulation of various biological processes, including cell differentiation, development, metabolism, and stress responses (Sun et al. 2018). In this study, we cloned *ZmbHLH36*, encoding a maize bHLH transcription factor. Bioinformatic analysis revealed that ZmbHLH36 contains the conserved domain characteristic of the bHLH family. Subcellular localization showed that ZmbHLH36 localizes to the nucleus. We also performed a preliminarily analysis of the biological functions of ZmbHLH36 in plant responses to abiotic stress using transgenic *Arabidopsis* lines.

Several studies on bHLH transcription factors in other plants have been reported. The maize bHLH transcription factor ZmbHLH91 may be involved in regulating plant tolerance to abiotic stress conditions such as high-salt, drought, and osmotic stress, based on the phenotypes of transgenic *Arabidopsis* lines expressing *ZmbHLH91* (Yue et al. 2022). The *Tamarix hispida* (*Tamarix chinensis*) transcription factor ThbHLH1 enhances abiotic stress tolerance by increasing osmotic potential and reducing ROS accumulation (Ji et al. 2016). In this study, our RT-qPCR analysis showed that *ZmbHLH36* is expressed in multiple maize tissues, with higher expression in roots at the three-leaf stage vs. the tasseling stage. This finding, combined with the results of previous transcriptome analysis, suggests that *ZmbHLH36* might be involved in regulating the growth and development of maize roots (Zhang et al. 2020). Moreover, abiotic stresses such as low-temperature, drought, and salinity, finding that they induced *ZmbHLH36* expression. The highest expression level in response to mannitol was observed in roots at 5 h. In response to cold and salt treatments, the expression levels were highest in roots at 2 h. For the dehydration treatment, the expression level of *ZmbHLH36* was highest in leaves at 24 h. These results suggest that *ZmbHLH36* functions in plant responses to abiotic stress. To test this hypothesis, we generated genetically modified *Arabidopsis* seedlings exogenously expressing *ZmbHLH36* and subjected them to high-salt, osmotic, and other abiotic stress factors. The seedlings expressing *ZmbHLH36* had longer roots than WT seedlings under abiotic stress, indicating greater tolerance of the stresses.

We also investigated the possible links between *ZmbHLH36* and the signaling pathways of two plant hormones: JA, because bHLH transcription factors play important, generally conserved roles in the signaling cascade triggered by this hormone (Goossens et al. 2017); and ABA, because it plays a crucial role in regulating plant responses to drought stress. AtAIB is a bHLH transcriptional activator involved in regulating ABA signaling in *Arabidopsis* (Li et al. 2007). The downregulation of *AtAIB* in *Arabidopsis* led to reduced sensitivity to ABA, while overexpressing *AtAIB* had the opposite effect. In the current study, we simulated phytohormone-mediated stress responses by growing *Arabidopsis* plants on medium supplemented with ABA and JA. Under these hormone treatments, transgenic *Arabidopsis* plants harboring *ZmbHLH36* had significantly longer roots compared to the control plants. We also subjected soil-grown plants to drought and high-salt stress and found that *ZmbHLH36* expression enabled *Arabidopsis* to withstand these stresses, resulting in greener leaves and healthier plants compared to the control. The increased stress tolerance of transgenic *Arabidopsis* expressing *ZmbHLH36* was further supported by the increased expression of the marker genes, reduced MDA content, and increased POD activity under stress conditions.

Our results indicate that ZmbHLH36 acts as a positive regulator of plant responses to high-salt, drought, osmotic, and phytohormone stress. Under stress conditions, ZmbHLH36 may regulate root growth and development, modulate membrane lipid peroxidation, stabilize the cell membrane system, and enhance antioxidant enzyme activity to alleviate the damage caused by drought and high-salt stress.

## Materials and methods

### Plant materials and growth conditions

Maize inbred line B73 was cultivated at the experimental station of Beijing Academy of Agriculture and Forestry Sciences (39°56′38.41″N, 116°16′57.18″E). Roots and young leaves from plants at the three-leaf stage, as well as roots, stems, leaves, tassels, and silks from plants at the tasseling stage, were collected from inbred line B73. Young embryos, harvested 1 week after pollination, were utilized for tissue-specific differential expression analysis. Root tissues from plants at different stages of growth (V6, V12, VT, and R3) were collected for RT-qPCR. For abiotic stress treatment, B73 maize seeds were placed in a growth chamber at 25 °C and a relative humidity of 70%. The seedlings were transferred to hydroponic containers and grown until the three-leaf stage. Subsequently, the seedlings were treated with a high salt concentration (placed in hydroponic containers containing 0.2 mol L^−1^ NaCl), osmotic stress (placed in hydroponic containers containing 0.2 mol L^−1^ mannitol), cold stress (at 4 °C), and dehydration stress (placed on absorbent paper for natural dehydration). The aboveground parts and roots were collected at 0, 1, 2, 5, 10, and 24 h of each treatment, rapidly frozen in liquid nitrogen, and stored at −80 °C.

### Bioinformatics analysis

The CDS of the transcription factor gene *ZmbHLH36* and the 2-kb sequence upstream of the transcription start codon (ATG) were obtained from the maize database (Ensembl Plants). The online tools SOPMA, THMMM, and Plant CARE were used to perform preliminary bioinformatics analysis of ZmbHLH36.

### RNA extraction and RT-qPCR

RNA extraction was performed using a FastPure Universal Plant Total RNA Isolation Kit (Vazyme Biotech Co., Ltd., Beijing) according to the manufacturer’s instructions. cDNA synthesis was carried out using a HiScript III RT SuperMix for qPCR (+ gDNA wiper) kit (Vazyme Biotech Co., Ltd., Beijing). Specific primers, including p*ZmbHLH36* RT-F, p*ZmbHLH36* RT-R for the target gene *ZmbHLH36*, and p*GAPDH* RT-F, p*GAPDH* RT-R for the maize reference gene *GAPDH*, were designed using the Primer-BLAST tool on the NCBI website and utilized for tissue-specific differential expression analysis (primer information can be found in Table S1). Real-time fluorescence amplification was performed using the BIO-RAD CFX96 Real-Time System qPCR instrument (Table S1). Each tissue sample included three biological replicates (separate experiments), and each biological replicate included three technical replicates (replicate samples). The data are presented as the mean ± standard error of three biological replicates. The 2^−∆∆CT^ method was used to obtain relative gene expression levels.

### Vector construction and transformation of *Arabidopsis*

The CDS of *ZmbHLH36* without the terminator sequence was amplified using the homologous arm primers p*ZmbHLH36* OE-F and p*ZmbHLH36* OE-R (primer information can be found in Table S1). The amplified CDS fragment was recombined into the expression vector pYBA1132 (pYBA1132 was kindly provided by Professor Fengping Yang from the Institute of Crop Sciences, Beijing Academy of Agriculture and Forestry Sciences), driven by the cauliflower mosaic virus (CaMV) E35S promoter, at the N-terminal region of the EGFP (enhanced green fluorescent protein) element, using the seamless cloning kit ClonExpress Ultra One Step Cloning Kit V2 from Vazyme Biotech Co., Ltd., Beijing. Transformed *Escherichia coli* DH5α competent cells were selected and propagated overnight in 5 mL liquid medium containing kanamycin (Kan^+^). The bacterial culture was then used for plasmid isolation, followed by digestion with restriction enzymes for verification. Correct plasmids were selected and sent to Sangon Biotech (Shanghai) Co., Ltd. for sequencing.

The sequenced overexpression vector was transformed into *Agrobacterium tumefaciens* strain GV3101. Single clones were selected for colony PCR and sequencing for verification. *Arabidopsis* (WT) inflorescences at the flowering stage were transformed with the vector using the floral dip method (Krenek et al. 2015). T_1_ generation seeds were harvested and air-dried for 1 week, followed by cold stratification for future use. T_1_ generation seeds were sterilized in 0.3% sodium hypochlorite solution for 15 min, rinsed with sterile water five to eight times, and sown on medium containing 0.02% Basta. After 8–10 days, surviving positive seedlings were transplanted to nutrient soil to obtain single T_1_ transgenic *Arabidopsis* plants. Genomic DNA was extracted from the leaves of T_1_ plants as a template for PCR analysis using the primers p*ZmbHLH36*-F and p*ZmbHLH36*-R. RNA was extracted from the leaves of T_1_ plants and reverse transcribed into cDNA as a template for RT-qPCR analysis. The expression levels were detected using the primers p*ZmbHLH36* RT-F, p*ZmbHLH36* RT-R, p*Actin1*RT-F, and p*Actin1*RT-R as internal controls. T_3_ generation homozygous plants were obtained from single plants with high expression and tested for subsequent experiments (Table S1).

### Root growth assay

To study the root growth of *Arabidopsis*, the seeds of transgenic and WT *Arabidopsis* were cultured on 1/2 MS agar plates for 7 days. Five germinated seedlings per line with the same growth status were selected and gently transferred to 1/2 MS agar plates supplemented with 150 mM NaCl, 200 mM mannitol, 50 μM ABA, or 100 μM JA. After 8 days of vertical growth, the root length of the seedlings was measured using ImageJ software (NIH, Bethesda, MD, USA).

### Drought and NaCl treatment of transgenic *Arabidopsis*

WT and transgenic *Arabidopsis* seeds were sterilized in 0.3% sodium hypochlorite solution for 10 min and sown on 1/2 MS medium. The plates were placed in the *Arabidopsis* growth chamber and incubated for 6 days. WT and transgenic *Arabidopsis* seedlings with similar root lengths and growth statuses were selected and transplanted into soil. Four seedlings were transplanted to each pot, with a total of nine pots per group. For the control group, the seedlings were watered normally. For the high-salt treatment, the seedlings were grown in soil for 25–30 days and irrigated with 300 mmol L^−1^ NaCl solution. Phenotypic data and physiological indicators were measured after 7–10 days. For drought treatment, the seedlings were grown in soil for 2 weeks, then deprived of water for 20–25 days. During this period, the stem height and fresh weight of *Arabidopsis* were measured using a ruler and analytical balance, respectively. The chlorophyll content of *Arabidopsis* was observed and analyzed, and the MDA content and POD activity were measured using physiological indicator kits.

### Subcellular localization of proteins

The subcellular localization vector was constructed as described above (for plant expression vectors). The pYBA1132-EGFP empty vector lacking the target gene was used as a negative control. The subcellular localization vector and empty vector, each at a concentration of 20 μg, were separately transformed into maize protoplasts derived from yellow maize seedlings. Following incubation at 25 °C under greenhouse conditions for 12 h, the green fluorescent signals were detected under a confocal laser-scanning fluorescence microscope (Nikon).

### Statistical analysis

The experiments were repeated three times, and the data are presented as the mean ± standard error of the mean (SEM). The experimental data were analyzed using GraphPad Prism software. In all analyses, *P* < 0.05 was taken to indicate statistical significance.

## Supplementary Information

Below is the link to the electronic supplementary material.

Supplementary file 1 (DOCX 1100 KB)

## Declarations

### Data availability

All data are available from the corresponding author upon reasonable request.
