# Supplementary material for: Heterologous expression of the maize transcription factor ZmbHLH36 enhances abiotic stress tolerance in Arabidopsis
Source: aBIOTECH. 2024 May 13;5(3):339–50. doi: 10.1007/s42994-024-00159-3 (PMC11399482; doi:10.1007/s42994-024-00159-3)
Supplement: Supplementary file 1 — (DOCX 1100 KB) [file 42994_2024_159_MOESM1_ESM.docx]

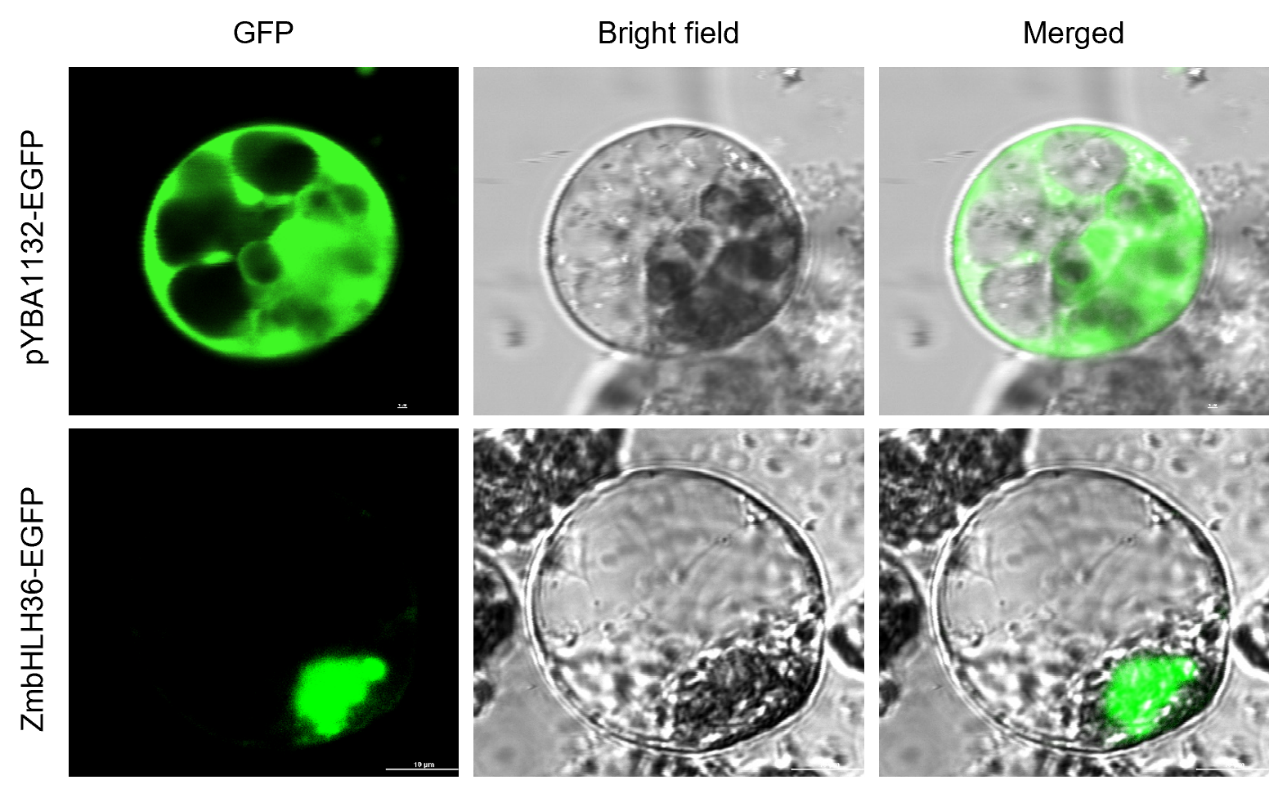


**Fig. S1 Subcellular localization of ZmbHLH36 protein in maize leaf protoplasts.**

The maize inbred line Z58 albino seedlings were transformed with pYBA1132-*ZmbHLH36*-*EGFP* using the PEG-Ca^2+^ mediated method. After 12 h of dark incubation, the transformed protoplasts were observed under a laser confocal microscope. GFP: Subcellular localization of GFP-labeled plasmids in maize protoplasts; Bright field: Maize protoplasts in the same field of view under bright-field microscopy; Merged: Overlay of bright-field and fluorescence images; pYBA1132-*EGFP*: Protoplasts transformed with the empty vector; pYBA1132-*ZmbHLH36*-*EGFP*: Protoplasts transformed with the target gene vector.


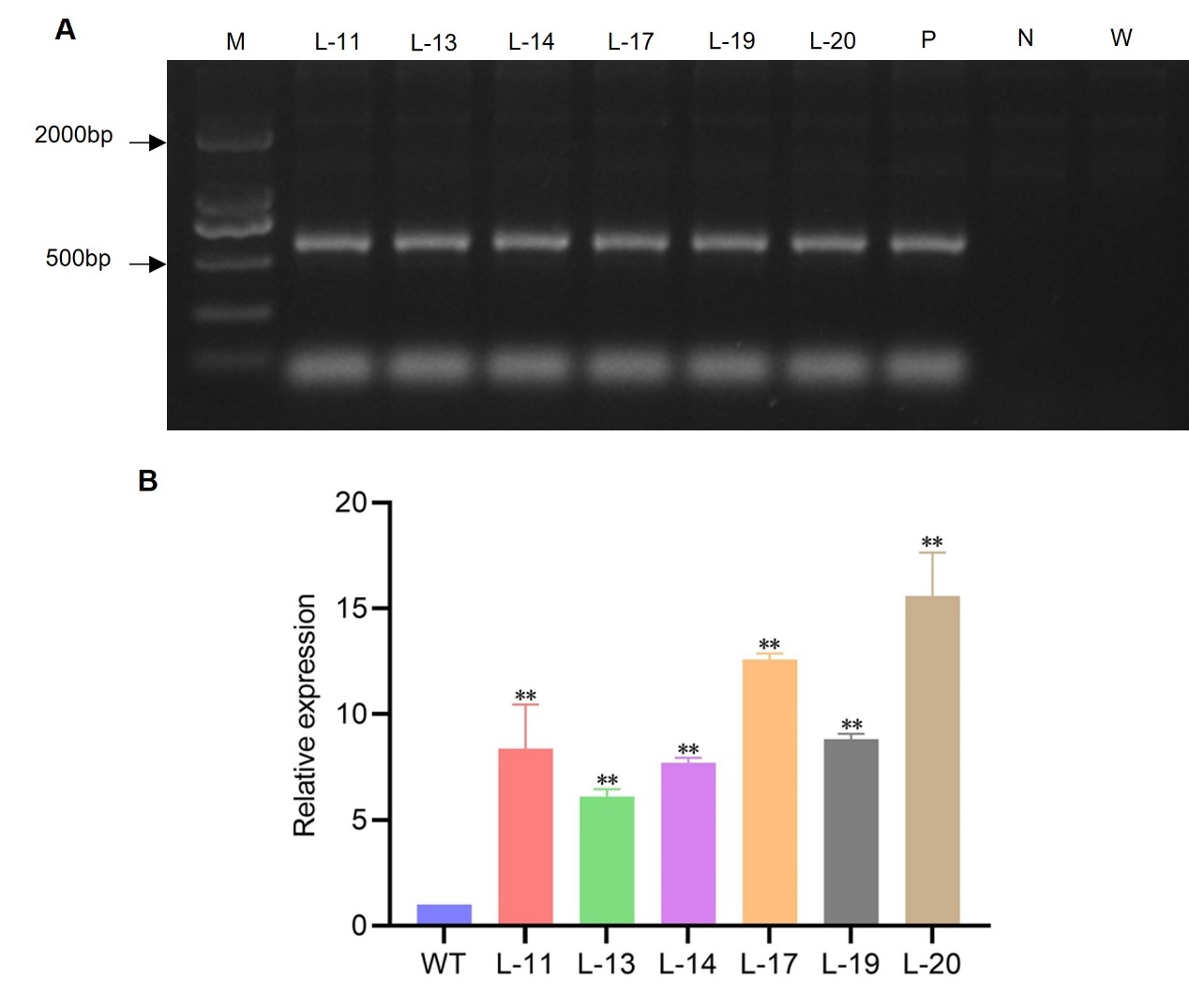


**Fig. S2 The T3 generation plants of transgenic *Arabidopsis* detection analyses by PCR and RT-qPCR.**

**A** PCR detection of T3 generation *Arabidopsis* plants. **B** RT-qPCR detection of T3 generation *Arabidopsis* plants. M: DL 2000 marker; P: Positive control; N: Negative control; W: Water control; WT: Wild-type *Arabidopsis*; L-11、L-13、L-14、L-17、L-19、L-20: T3 generation *Arabidopsis* lines; **: *P*<0.01. Leaves DNA were extracted from T1 generation *Arabidopsis* plants, which was screened on 0.02 % Basta plates, for PCR detection. Independent transformation events were obtained, and after subsequent propagation of positive lines, T3 generation seedlings leaves were collected for PCR detection and RT-qPCR identification. Homozygous transgenic *Arabidopsis* seeds carrying the *ZmbHLH36* gene were obtained. As shown in Figure S2A, negative control and water control showed no bands, while transgenic plants and positive control showed purposeful bands that were consistent. Therefore, six transgenic *Arabidopsis* lines carrying the target gene were obtained (named as L-11, L-13, L-14, L-17, L-19, L-20). RT-qPCR analysis of the target gene in these six lines showed significantly higher expression levels compared to the wild-type lines (Fig. S2B). Among them, lines L-11, L-17, and L-20 with the highest expression level were selected for subsequent phenotype analysis experiments.

**Table S1. The list of primers used in this article**

| **Primer Name** | **Sequences (5'-3')** | **Usage** |
| --- | --- | --- |
| p*ZmbHLH36* RT-F | GGTGAAGAACACGGAGGAGG | RT-qPCR |
| p*ZmbHLH36* RT-R | CTGGTCACCCCGCTGC |  |
| p*GAPDH* RT-F | CCCTTCATCACCACGGACTAC |  |
| p*GAPDH* RT-R | AACCTTCTTGGCACCACCCT |  |
| p*Actin1* RT-F | GGTAACATTGTGCTCAGTGGTGG |  |
| p*Actin1* RT-R | AACGACCTTAATCTTCATGCTGC |  |
| p*ZmbHLH36*-F | GAACGAGTACCTCTCCGTGC | RT-PCR |
| p*ZmbHLH36*-R | GCTGAACTTGTGGCCGTTTA |  |
| p*ZmbHLH36* OE-F | GAAATCACCAGTCGGTACcATGGCGCTGGAAG |  |
| p*ZmbHLH36* OE-R | CCTTGCTCACCATGGTACCCTACGCGGCTAGT |  |
